# Supplementary material for: The independent factors associated with oxygen therapy in COVID-19 patients under 65 years old
Source: PLoS One. 2021 Jan 22;16(1):e0245690. doi: 10.1371/journal.pone.0245690 (PMC7822310; doi:10.1371/journal.pone.0245690)
Supplement: Attachment — Submitted filename: response to reviewers.docx [file pone.0245690.s002.docx]

Dear Editor,

Re: Manuscript ID PONE-D-20-25061

We would like to thank *plos one* for giving us the opportunity to revise our manuscript.

We thank the reviewers for their careful reading and thoughtful comments on previous draft.

We have carefully taken their comments into consideration in preparing our revision, which has resulted in a paper that is clearer, more compelling, and broader. The following summarizes how we responded to reviewer comments.

Thanks for all help.

Best wishes,

Dr. Liang Zongan

Corresponding Author

Review Comments to the Author

Reviewer #1: Ni Yue-Nan et al. submitted a manscript about the factors associated with the need of start oxygen therapy in people with SARS-CoV-2 infection under 65 years old. Many issues are present:

Abstract
The abstract is well structured. However, some sentences present mistakes. For example, the sentence “The proportion and number of young novel corona virus disease 2019(COVID-19) hospitalized patients might increase as many countries reopen.” should be reformulated.
833 should be written in letters.
The sentence “29.4% of them have coexisting condition.” is unclear.
“p” should be written in italic style
Many conjunctions are misused.

**Answer：**

Thanks a lot for your careful reading. We have revised our manuscript item by item.

Introduction
The sentence “Meanwhile, most of the people older than 65 years old could continue to stay at home” has no source.

**Answer:**

Thanks for your advice. We have restructured this sentence and cited the resources（<https://www.cdc.gov/mmwr/volumes/69/wr/mm6939e4.htm?s_cid=mm6939e4_x>）

I suggest adding some more information about the COVID-19 and its symptoms in the first part of the introduction. My suggestion is to write that COVID-19 is caused by a virus, SARS-COV-2, and its major symptoms are fever, cough, and dyspnea, and minor symptoms are alteration of the smell and taste, gastrointestinal symptoms, headache, and cutaneous manifestations. You could read and use these articles: https://doi.org/10.1002/hed.26269, https://doi.org/10.1002/hed.26204, https://doi.org/10.26355/eurrev_202007_22291, https://doi.org/10.1016/S1473-3099(20)30402-3, https://doi.org/10.1016/S0140-6736(20)30183-5, <https://doi.org/10.1111/jdv.16669>).

**Answer:**

Thanks a lot for your advices. We have added more information about the COVID-19 and cited these articles.

Methods
Which test to evaluate the probability distribution have you used? Please, add this information
**Answer:**

Thanks for your meaningful suggestion. We used Z test to test the distribution, and these details have been added in our manuscript.

The authors wrote that oxygen therapy was prescribed only when emergency signs were present. Did 63.1% of your patients have one of those emergency signs?. Please comment.
**Answer：**

Thanks for your careful reading. Our clinical practice strictly followed the guidelines which indicated that: when emergency signs (obstructed or absent breathing, severe respiratory distress, central cyanosis, shock, coma, or convulsions) was observed, oxygen therapy should be administrated. However, due to the study design of our study, all the data was retrospectively extracted from the medical records, we could not sure whether all the patients received oxygen therapy due to they have one or more emergency signs. We have strengthened this in the limitation part of our study

Results
The authors said “The proportion of current alchohol abuse patients was equal between the two groups (9.7% vs. 10.7%, p=0.626)”. There is a point percentage of difference, so the proportion is not equal. Please fix it, and correct the word alcohol.
**Answer:**

Sorry for our confusing convey. We have revised the sentence as the proportion of current alcohol abuse patients was not significantly different between the two groups (9.7% vs. 10.7%, p=0.626).

In the methods, the authors wrote that fever was defined as the body temperature ≥ of 37.3°C. In the results, they wrote 37.5. Please verify which one is correct.
**Answer:**

Thanks a lot for your careful reading. And sorry for our typo. It is 37.5 °C. We have revised relevant sentence in our manuscript.

In the “Laboratory indices” subsection, all the Unit of measurement are missing. Please add them.
The Neutrophils / Lymphocytes ratio results to be 4.85± 6.89. A ratio can not be negative. The same goes for ALT, AST, D-dimer, urea, creatinine, and glucose. Probably you should use the median and not the mean. Please verify the distribution of all laboratory tests and use mean or median as appropriate.
**Answer：**

Thanks a lot for your careful reading. We have retested whether these variables and used median instead of mean for those without standard normal distribution.

The authors reported that 15 patients in the oxygen therapy group died. No data about deaths in the other group are present. Please add it.
**Answer:**

Thanks a lot for your careful reading. No death in other group. We have added it.

Do the authors know the length of hospitalization for the two groups? There is a difference? Please comment.
**Answer:**

Thanks for your meaningful advice. We have calculated the length of hospitalization for the two groups. The ones who have received oxygen therapy have longer hospitalization (16.00 vs. 14.50, *p*=0.002). We have added discussion about the difference in length of hospitalization between the two groups.

Discussion

The author wrote, “The successful rate of oxygen therapy is high and the risk for comorbidities is low.”; it is not clear what “the risk for comorbidities is low” means. Please reformulate the sentence.
**Answer：**

Sorry for our confusing convey. We means that the risk for young Covid 19 patients developing complications during hospitalization is relatively low. We have revised our manuscript.

Tables
In table one, in the section about symptoms and comorbidities, the authors wrote “yes or no” in all symptoms. It is not clear what “yes or no” means. The authors should report in the subtitle which tests they used to calculate the p-value for each variable.
The Neutrophils / Lymphocytes ratio results to be 4.85± 6.89. A ratio can not be negative. The same goes for ALT, AST, D-dimer, urea, creatinine, and glucose. Probably you should use the median and not mean. Please verify the distribution of all laboratory tests and use mean or median as appropriate. For the urea, creatinine, glucose, please correct the Unit of measurement: mMol/L.
Furthermore, I suggest adding a column with the characteristics of the whole cohort.

About table 2, why urea was included in the multivariate analysis if the p-value was 0.127? Please comment.

**Answer:**

Sorry for our carelessness.

1. We have revised changed the convey “yes or no”.
2. We have added the relevant information about which test we have used in the Method part and indicated in the Table 1 which test was used.
3. We have retested whether these variables and used median instead of mean for those without standard normal distribution.
4. We have added the column with the characteristics of the whole cohort.
5. We have deleted the “Urea” in the multiple logistic regression and revised the table 2. Fortunately, all the results remained unchanged.

Table 3: it is not clear what “yes or no” means.
Answer：

Sorry for our confusing expression. We have changed the way of conveying. Hope it is clear this time.

General comments
There are many English mistakes about the use of prepositions, conjunctions, and tense. I believe that a Native speaker should revise the manuscript.

Answer:

Thanks for your meaningful advice. We have turn to American Journal Expert, which is a professional English editing company, for help. They have helped us editing our language.

All number ≤ 10 should be written in letters. All numbers at the sentence beginning should be written in letters (example, “833 patients”, and “526” in the results section).

Answer:

Thanks a lot for your careful reading. We have revised all the relevant numbers.

All abbreviations should be written entirely in the first appearance in the text. Please check all of them.

Answer:

Thanks a lot for your advice. We have checked again and revised our manuscript.

Reviewer #2: Dear Authors,
I would like to thank you for the paper you submitted and I had the chance to review. I found quite interesting the main background of the study – the importance to have data on <65 years/old COVID-19 patients to relocate resources now that younger people will be more exposed to SARS-CoV-2 for reopening of public services. The study you submitted is well-designed and inclusion criteria well-established on basis of literature, but I have a major concern I will explain as follow: in “methods” paragraph you say that the need for oxygen therapy was determined by “emergency signs” clinically established, with no mention to arterial blood gases parameters. I would like you to further explain this peculiar decision, because I found incorrect to diagnose lung failure or hypoxemia without ABG. Many recent works found properly an independent association between lower P/F ratio (Horowitz ratio) at the admission and prolonged hospitalization.

Answer:

Thanks a lot for your careful reading and meaningful advice. We totally agree with you that without ABG, clinicians can not diagnose hypoxemia with a 100% correct rate.

As mentioned above, our study was retrospective study, we could not sure the reason for oxygen therapy in every patients, although our clinicians strictly followed the guideline published by the WHO. It is not mentioned that ABG is a remark or indication for oxygen therapy in the guideline. We guess the reason was that：since the strong infectiousness and extraordinary quick development of the disease, it is unlikely to do the ABG for every patients before oxygen therapy. Obviously, in the special pandemic, ABG is not very convenience and feasible as usual. Moreover, lack of clinicians during the pandemic may be another reason.

However, we think your concern was totally right and reasonable. We have strengthened it in the limitation part of our manuscript.

I have a minor concern about association with laboratory findings, because many data were missing for many of them and such discrepancy may have affected the solidity of statistical association. However, independent association with complications and increased death rate and lower lymphocyte count retraces experiences described in other countries, such as you may read here 10.26355/eurrev_202007_22291. Moreover, some of the blood count alterations you identified are peculiar for COVID-19 rather than other pneumonia, as you may read here <https://doi.org/10.3855/jidc.12879>.

Answer:

Thanks a lot for your meaningful advice. We should admit that the missing of laboratory findings will definitely affect our results. The results of our study have something in common with the previous published papers such as the two listed. However, the population of our study was special, younger, and otherwise, we have larger sample size.

We have added the discussion about the differences between our laboratory findings and other published papers, and also the influence of missing laboratory data in the limitation part.

In the end, if chest distress and dyspnoea are more obviously associated with respiratory failure, association with fever is less understandable. I would like you to expand more this peculiar finding, especially because no significant difference on incidence of fever was found between the two patients groups.

Answer:

Thanks a lot for your meaningful advice. We have expanded the discussion about fever in our manuscript.

The language used is generally appropriate and the paper is written in acceptable English, with few typos and many grammar mistakes I suggest you to check.

Answer:

Thanks for your meaningful advice. We have turn to American Journal Expert, which is a professional English editing company, for help. They have helped us editing our language.
