# Supplementary material for: The independent factors associated with oxygen therapy in COVID-19 patients under 65 years old
Source: PLoS One. 2021 Jan 22;16(1):e0245690. doi: 10.1371/journal.pone.0245690 (PMC7822310; doi:10.1371/journal.pone.0245690)
Supplement: Attachment — Submitted filename: response to reviewers.docx [file pone.0245690.s003.docx]

Dear Editor,

Re: Manuscript ID PONE-D-20-25061

We would like to thank *plos one* for giving us the opportunity to revise our manuscript.

We thank the reviewers for their careful reading and thoughtful comments on previous draft.

We have carefully taken their comments into consideration in preparing our revision, which has resulted in a paper that is clearer, more compelling, and broader. The following summarizes how we responded to reviewer comments.

Thanks for all help.

Best wishes,

Dr. Liang Zongan

Corresponding Author

Review Comments to the Author

Please use the space provided to explain your answers to the questions above. You may also include additional comments for the author, including concerns about dual publication, research ethics, or publication ethics. (Please upload your review as an attachment if it exceeds 20,000 characters)

Reviewer #1: I have reread the manuscript carefully. The authors have modified the manuscript as suggested. However, some issues are still present.

The sentence in the “Symptoms and signs at admission” paragraph is hard to read; I suggest modifying it specified which values are from the oxygen group and the other group.

Answer：

Thank you for your advice. We have revised it.

Furthermore, the authors wrote, “A higher respiratory rate of 20 (19-21) vs. 20 (19-20) times/minute (p=0.005)”; I believe there is a typo in the number because it is odds that the number is equal, but the p-value is significative; then, I suggest to specify the group.

Answer:

Thanks for your careful reading. We have checked. The number is right as well as the *p* value. Although the median and Q1 were the same in the two groups，the Q3 was obviously higher in the oxygen therapy group. Which means, the percentage of patients who have RR>20 was higher in the oxygen therapy group.(we have provide the detail in the Table 1). Because the RR was not normal distributed, we used the nonparametric test, which calculated the rank of the RR value of each patient in the two groups. Since higher percentage of the patients who had RR>20 in the oxygen therapy group was found, the mean rank in the oxygen therapy group was higher and the p-value was significative.

Also, in the laboratory findings, it is unclear which values are from one group and another. I suggest to specified, maybe at the start of the paragraph.
Answer:

Sorry for our confusing expression. We have revised this paragraph and tried to make it more clear.

A recent study (https://doi.org/10.1111/eci.13427) found that De Ritis ratio was a predictor of hospital mortality in COVID-19 patients. In your paper, there was a significant difference regarding ALT and AST values in the two groups. Furthermore, in univariate analysis, they showed a high OR, not confirmed in the multivariate analysis. Could you verify if the De Ritis ratio was higher in the oxygen group? I believe that it could improve the value of your work.

Answer：

Thank you for your meaningful advice. We have tested the ratio of AST/ALT, however, no significant between the two groups has been found. Moreover, we have added discussion about the ratio of AST/ALT.

Are you sure that the urea level (4.61±4.34) has a normal distribution? Please verify it.
Answer:

Sorry for our carelessness. We have revised it and relevant part in the manuscript.

It is not clear if it is a mean or a median regarding the length of hospitalization. Please specified it, and add the standard deviation or IQR, as appropriate.
Answer:

Thank you for your careful reading. We have revised our manuscript accordingly.

In table 1, in the variable column, regarding the non-continuous variable (e.g. gender, smoker), the authors wrote that they are expressed in “no./total no. (%)”, However, the denominator is missing. In table 3, instead, it is present. Please level out this aspect.
Answer:

Thank you for your careful reading. We have uniform our expression.

Table 2. In the column of the multivariate p-value, the p-value is expressed without the zero before the dot. Please add the zero to level out the column with the manuscript. Furthermore, “p” in table 1 is written in capital letters. Please modify it; it should be written in lowercase and in italic style.

Besides, you should add, both in “Univariable” and in “Multivariable”, “ OR (95%CI), and explain the meaning of these abbreviations in the subtitle.

Answer:

Thank you for your advice, we have revised the Tables item by item.

I suggest modifying “T”, with “Body temperature”.

Table 3. I suggest removing “all” and “N=526”. I suggest modifying the caption in “Treatment details and complications in 526 patients received oxygen therapy”. Furthermore, in the caption the explanation of ARDS is missing.

Answer:

We have revised the Table 3 accordingly.
